# Vibrio vulnificus Bacteremia With Compartment Syndrome: A Case Report and Characterization of the Isolate

**DOI:** 10.7759/cureus.72217

**Published:** 2024-10-23

**Authors:** Muhammad Areeb Ashfaq, Hajira Z Malik, Yazan Fahmawi, Teresa Barnett, Patricia Hacker, Jessica Jones, Madison McGough, Amanda Tuckey, Victor Solodushko, Jonathon Audia, Brian Fouty

**Affiliations:** 1 Department of Internal Medicine, University of South Alabama, Mobile, USA; 2 Division of Hematology and Medical Oncology, University of South Alabama, Mobile, USA; 3 Division of Cardiovascular Medicine, University of South Alabama, Mobile, USA; 4 Division of Pulmonary and Critical Care Medicine, University of South Alabama, Mobile, USA; 5 Clinical Microbiology Lab, University of South Alabama, Mobile, USA; 6 Division of Seafood Science and Technology, United States Food and Drug Administration, Dauphin Island, USA; 7 Department of Immunology and Microbiology, University of South Alabama, Mobile, USA; 8 Center for Lung Biology, University of South Alabama, Mobile, USA; 9 Department of Pharmacology, University of South Alabama, Mobile, USA

**Keywords:** compartment syndrome, fasciotomy, martx toxin, vibrio vulnificus, virulence-correlated gene

## Abstract

*Vibrio vulnificus* is a Gram-negative, curved, rod-shaped organism that can cause sepsis due to either gastroenteritis when ingested (usually via raw oysters) or skin infections when introduced into cuts or abrasions. Found in estuarine waters (coastal waters where fresh water from streams mixes with salt water from the ocean resulting in water of intermediate salinity (i.e., brackish water)), it most commonly causes clinical disease in individuals with cirrhosis, diabetes, or other immunocompromised states. Here, we describe the case of a 59-year-old male, with known heavy alcohol use and hypertension, who presented to the emergency department with acute cellulitis and compartment syndrome of his right leg due to *V. vulnificus*. He had a rapidly fatal course despite aggressive surgical and medical care. We describe his clinical course, identify sequence relatedness, and detail the characterization of the toxins expressed by his isolate. Early consideration of *V. vulnificus* infections is important in reducing morbidity and mortality in high-risk patients; however, even with early detection and treatment, mortality remains high. Improving survival will require a better understanding of the host, bacteria, and environmental factors that determine clinical outcomes. This will require improved coordination between clinicians, microbiologists, and basic scientists.

## Introduction

*Vibrio vulnificus *is a Gram-negative, rod-shaped bacterium with the highest case mortality rate of all food-borne pathogens. It has a rapid induction period that is often less than 36 hours. It is found more commonly in warm estuarine waters and has the potential to cause wound infections, gastroenteritis, and bacteremia [1,2]. In the aftermath of Hurricane Ian, Florida reported at least 65 cases of *V. vulnificus *infection, a trend often observed after a hurricane [3]. Individuals with certain comorbidities (particularly cirrhosis or diabetes) are at the highest risk, although serious infection can occur in their absence [4]. The major environmental risks include ingestion of shellfish harvested from, or wound exposure to, brackish water [1].

Virulence is mediated primarily by contact-dependent secretion of a toxin known as the MARTX (multifunctional autoprocessing repeats-in-toxin) toxin. The MARTX toxin first forms pores in eukaryotic cell membranes and then delivers its effector domains directly into the host cytoplasm, resulting in rapid cytoskeletal rearrangements and cytolysis of epithelial cells, endothelial cells, erythrocytes, and macrophages [5-7]. The MARTX type is based on the cadre and arrangement of its effector domains which differs widely between isolates [7,8]. Rosche and colleagues also identified sequence differences in a virulence-correlated gene (vcg) locus that discriminated between *V. vulnificus *strains obtained from clinical sources (human infection) (vcgC, Lineage 1) from those isolated from environmental sources (mainly obtained by screening shellfish) (vcgE, Lineage 2) [9]. Based on these putative *V. vulnificus* virulence determinants, we present this case report and characterization of the culprit strain isolated from the patient. Further, we report on the relatedness of the whole genome sequence of this isolate to other *V. vulnificus.*

## Case presentation

A 59-year-old male with known heavy alcohol use and hypertension presented to the emergency department with right lower extremity pain of a few hours of onset. He worked on the shoreline of Mobile Bay in brackish water and his legs had been exposed during the days before his symptoms. Upon admission, he was tachypneic (22 breaths/minute) and tachycardic (105 beats/minute) and appeared acutely ill. His right lower extremity was erythematous with mottling and blistering and the leg was tender to palpation. His initial arterial blood gas showed a pH of 7.14, pCO_2_ of 39 mmHg, pO_2_ of 98 mmHg, and HCO_3_ of 13 meq/L. Additional laboratory workup showed renal injury (creatinine: 2.30 mg/dL; normal range: 0.7-1.30 mg/dL) and elevation of liver-associated enzymes (alanine transaminase: 130 U/L (normal range: 14-59 U/L); aspartate transaminase: 97 U/L (normal range: 15-37 U/L) and bilirubin (1.6 mg/dL; normal range: 0-1.0) and elevated lactate (8.6 mmol/L). His Laboratory Risk Indicator for Necrotizing Fasciitis (LRINEC) score was 4 (low).

There was initial concern that the patient had a Group A Strep soft tissue infection with streptococcal toxic shock syndrome and he was treated with (renally dosed) vancomycin, clindamycin, and cefepime; doxycycline was added to cover *V. vulnificus* after his water exposure history was obtained. Due to the presence of tense skin in his right lower extremity, pain with dorsiflexion of the foot, and decreased posterior tibial and dorsalis pedis pulses, an immediate surgical consult was requested for possible compartment syndrome. The patient underwent emergent fasciotomy and debridement of his right leg and foot. Extensive edema with definite signs of compartment syndrome was observed, but there was no evidence of necrotizing fasciitis. Within 24 hours, blood cultures were positive for *V. vulnificus* which was sensitive to cefepime, doxycycline, and levofloxacin. Despite antibiotics, vasopressors, and intensive medical care, the patient developed refractory septic shock and died within 48 hours of presentation.

*V. vulnificus *isolate characterization

The patient’s blood cultures were processed by the University Hospital Clinical Microbiology Laboratory and *V. vulnificus* was isolated and identified to be the causative agent. This isolate was named AFM1 and cryopreserved at -80°C for the analyses described. This isolate was compared to previously validated Lineage 1 clinical isolates (vcgC) which were isolated from blood in patients from either Texas or Georgia and Lineage 2 environmental isolates (vcgE) which were isolated from oysters in Texas or Louisiana.

For vcg-locus typing of our patient isolate (AFM1), we followed the protocol described by Rosche and colleagues with the following modifications [9]. All *V. vulnificus *isolates (Table 1) were routinely propagated on trypticase soy agar containing 5% sheep red blood cells (TSB) and lysogeny broth (LB) liquid medium. A single colony was picked from a fresh TSB overnight culture into 2 mL of LB and cultured with aeration at 37°C until the culture was faintly turbid (two to three hours post-inoculation). The entire culture was harvested by centrifugation (5,000 ×g, five minutes, ambient temperature). The supernatant was discarded, the bacterial pellet suspended in 0.5 mL sterile water, and then incubated at 95°C for 10 minutes. The lysed culture was agitated on a vortex mixer and the debris was removed by centrifugation (5,000 ×g, five minutes, ambient temperature). Amplification reactions (20 μL) were prepared by adding bacterial lysate (1 μL) to GoTaq Green Master mix (Promega M7123) containing forward and reverse primer sets (0.1 μM each) specific for vcgE or vcgC, as described [9]. Thermocycling reaction conditions were 98°C for 30 seconds, (98°C for 10 seconds, 50°C for 15 seconds, 72°C for 30 seconds) ×40 cycles, 72°C for 10 minutes, and 10°C hold. Reactions were resolved by agarose gel electrophoresis (2% agarose II in 0.8 × TAE running buffer, 160 V, 30 minutes), and images were captured using a BioRad ChemiDoc gel documentation system.

**Table 1:** The list of V. vulnificus strains with their NCBI sample numbers (where available) that were used to generate the phylogenic tree in Figure 3. NA = not available; - = no signal detected on polymerase chain reaction (PCR); + = positive signal detected on PCR

Strain ID	Isolation date	State	Source	vcgE	vcgC	Analysis	NCBI BioSample #
AFM1	2-Oct-2022	AL	Clinical; Blood	-	+	WGS, MARTX	SRR29234674
ORL 8074	1995	TX	Clinical; Blood	-	+	MARTX	NA
CMCP6/Y016	NA	NA	NA	NA	NA	MARTX	NA
MO6-24/0	NA	NA	Clinical	NA	NA	MARTX	NA
FLA8869	1996	TX	Clinical; Blood	-	+	MARTX	NA
BCW_3431	March 14, 2007	TX	Environmental; oyster	+	-	WGS	SAMN02368403
BCW_3433	May 28, 2007	LA	Environmental; oyster	+	-	WGS	SAMN02368404
BCW_3446	July 30, 2007	LA	Environmental; oyster	+	-	WGS	SAMN02368414
BCW_3447	August 13, 2007	FL	Environmental; oyster	+	-	WGS	SAMN02368415
BCW_3434	June 19, 2013	LA	Environmental; oyster	-	+	WGS	SAMN02368405
BCW_3473	November 2, 2012	AL	Environmental; oyster	+	-	WGS	SAMN02368437
BCW_3486	July 7, 2006	AL	Clinical; other	-	+	WGS	SAMN02368444
BCW_3487	September 22, 2006	AL	Clinical; other	+	-	WGS	SAMN02368445
BCW_3488	April 18, 2007	MS	Clinical; blood	+	-	WGS	SAMN02368446
BCW_3489	April 17, 2007	TX	Clinical; blood	-	+	WGS	SAMN02368447
BCW_3491	May 16, 2007	TX	Clinical; blood	-	+	WGS	SAMN02368449
BCW_3502	November 10, 2007	GA	Clinical; blood	-	+	WGS	SAMN02368458

**Figure 1:**
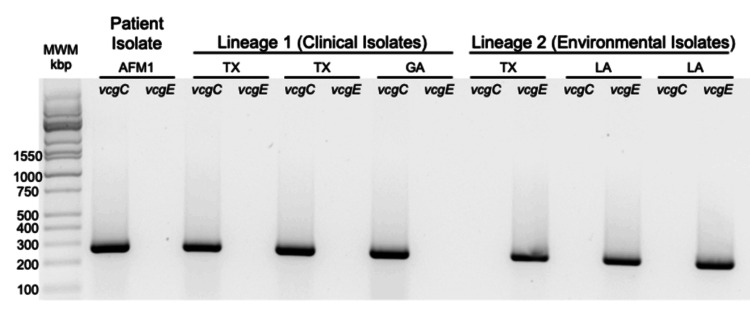
The patient’s V. vulnificus strain (AFM1) gave a polymerase chain reaction amplification profile pattern similar to V. vulnificus strains of known clinical origin (Lineage 1, vcgC). A single bacterial colony was inoculated into LB medium, grown for three hours at 37°C with aeration, and then lysed by heating. The resulting crude DNA preparation was used as a template for amplification with forward and reverse primer sets that were specific for either clinical (vcgC) or environmental (vcgE) strains of V. vulnificus. Amplification products were resolved by gel electrophoresis. The known clinical and environmental isolates are denoted by state of origin (TX = Texas; GA = Georgia, LA = Louisiana). MWM = molecular weight marker in kbp

The gel shown in Figure 1 demonstrates that the *V. vulnificus* isolate from the patient (AFM1) yielded a polymerase chain reaction (PCR) amplification product corresponding to Lineage 1 (vcgC).

For sequencing analysis of the MARTX from our patient isolate (AFM1), we followed the gene amplification protocol described by Kwak et al. [8] The primer set used amplifies the region encoding the MARTX effector domain. The resulting amplicon was verified by agarose gel electrophoresis and subsequently cloned using a Zero Blunt TOPO cloning kit (Invitrogen 450245). Six individual clones were selected for verification by restriction endonuclease digestion followed by whole plasmid sequencing (Oxford Nanopore, Eurofins Genomics). The resulting sequence was compared against known *V. vulnificus* MARTX toxin effector regions in the NCBI sequence database and aligned using CLUSTAL Omega.

**Figure 2:**
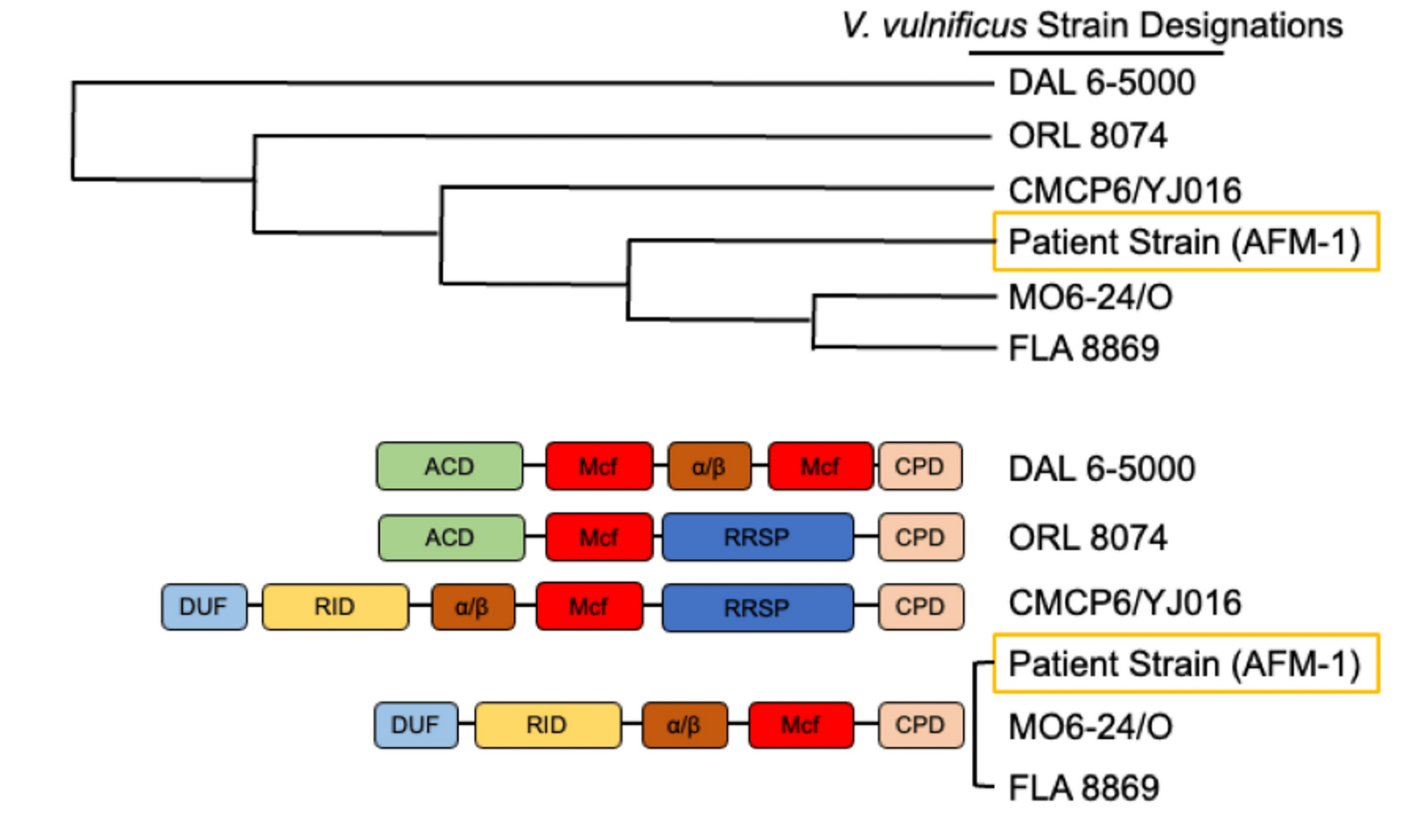
The sequence of the MARTX toxin obtained from this patient’s strain of V. vulnificus (AFM1) indicated that it was of the M type and contained four effector regions and a cysteine protease, similar to other strains that had been isolated from the Gulf Coast. The primer set used amplifies the region encoding the MARTX effector domain. Six individual clones were selected for verification by restriction endonuclease digestion followed by whole plasmid sequencing. The resulting sequence was compared against known V. vulnificus MARTX toxin effector regions in the NCBI sequence database and aligned using CLUSTAL Omega. The top panel shows an unrooted cladogram (neighbor-joining tree without distance corrections) and the bottom panel is an illustration of MARTX toxin effector domains (adapted from Kwak et al.). DUF = domain of unknown function; RID = Rho-inactivating domain; α/β = hydrolase; Mcf = makes caterpillar floppy; RRSP = Ras-Rap1-specific endopeptidase; ACD = actin-crosslinking domain; CPD = protease

Figure 2 shows a schematic of the MARTX effector domain of this patient’s isolate (AFM1) compared to other clinical isolates from around the world. The patient’s MARTX toxin was of the M-type and comprised four effector domains and a cysteine protease (which cleaves the effector domains into their subunits once they are introduced into the host cell). Its sequence was most similar to clinical strains originating from the Gulf Coast region (Mobile and Florida).

**Figure 3:**
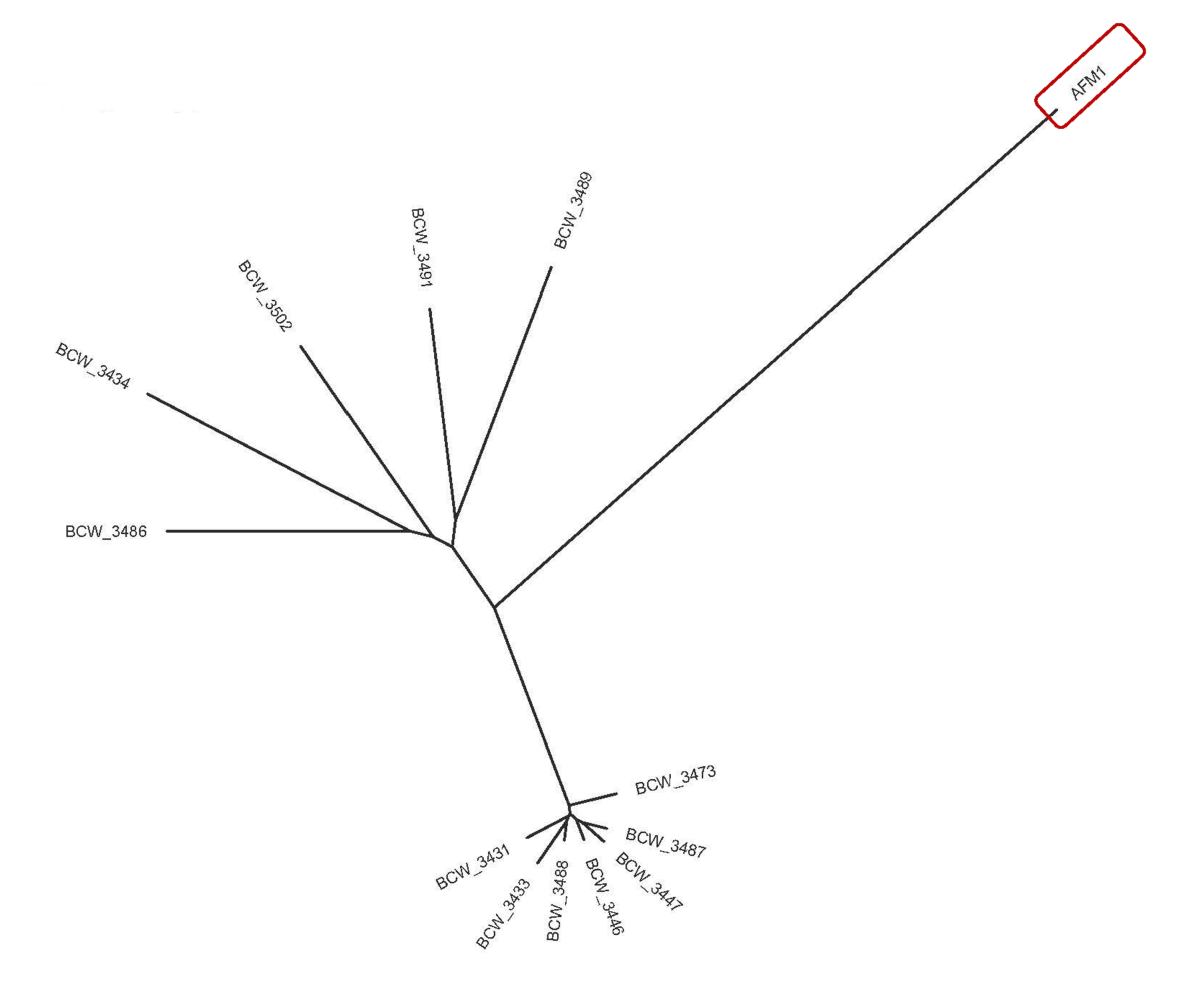
The whole genome sequence from the patient isolate (AFM1) indicated with a red box was compared to five vcgC and six vcgE previously sequenced isolates from clinical or environmental (oyster) sources and with sequence data publicly available (Table 1). The phylogenetic tree is based on an SNP distance matrix using a neighbor-joining algorithm. The known isolates are denoted by isolate ID (from NCBI). The patient isolate (AFM1) was slightly more related to the environmental (oyster) isolate cluster than the clinical isolate cluster. The isolate was submitted to the NCBI and its sequence number is SRR29234674.

For whole genome shotgun sequencing of AFM1, the isolate was streaked to trypticase soy agar and incubated overnight. A single colony was picked into tryptic soy broth and incubated overnight at 35 ± 2°C. A 1 mL aliquot of the culture was pelleted by centrifugation (10,000 ×g, two minutes, ambient temperature). DNA was extracted from the resultant pellet using the DNeasy Blood and Tissue Kit (Qiagen, Germantown, MD) with final elution in Buffer EB. The sequencing library was prepared using DNA Prep Tagmentation (Illumina, San Diego, CA) and Nextera CD Indexes (Illumina). The library was sequenced using v3 chemistry on a MiSeq (Illumina). The sequencing of comparative strains was conducted by the Weimer lab at the University of California - Davis through the 100K Pathogen Genome Project, as previously described [10,11]. FastQ sequences of all isolates were imported into GalaxyTrakr (galaxytrakr.org). The paired collection of FastQ files was trimmed using Trimmomatic (version 0.39) [12]. The trimmed collection was assembled using SPAdes (version 3.11.1), quality checked with QUAST (version 4.6.3), assemblies selected using Select Best (version 0.1.0), and then followed the SNP Pipeline workflow. The SNP matrix, fasta output was used as input for Join Neighbors rapidly with RapidNJ (version 2.3.2) [13]. FigTree (version 1.4.4) was used to create the phylogenetic tree. Figure 3 is a phylogenetic tree based on the SNP distance matrix. The patient isolate (AFM1) was less related to any of the comparison strains than they were to each other (SRR for the AFM1 Vibrio sequence is SRR29234674).

## Discussion

This patient presented with acute cellulitis with compartment syndrome due to* V. vulnificus* which rapidly progressed to septic shock. He succumbed to multi-organ failure within 48 hours of admission.

Three points are worth highlighting in this case*. V. vulnificus *wound infections can present as compartment syndrome in the absence of necrotizing fasciitis.* V. vulnificus *classically gains entry either through the gastrointestinal tract following ingestion of shellfish (oysters mainly) or through breaks in the skin following exposure to brackish water.* V. vulnificus* skin infections frequently cause necrotizing fasciitis, and for this reason, amputations (the definitive treatment for necrotizing fasciitis) are one of the more common complications associated with severe *V. vulnificus *wound infections [7,14,15]. The patient described here had cellulitis and compartment syndrome due to *V. vulnificus*, but not necrotizing fasciitis, a surgical observation that was subsequently confirmed by histology. This is similar to a published report by Hui et al. in 1999 who described a cirrhotic patient with compartment syndrome as the initial manifestation of *V. vulnificus* bacteremia who died of multiorgan failure and hepatorenal syndrome without developing necrotizing fasciitis [16]. However, this is in contrast to reports from Miron et al. and Leechavengvongs et al. who described patients with compartment syndrome who were subsequently found to have necrotizing fasciitis due to *V. vulnificus* at surgery [14,17]. The patient described in this report had acute cellulitis and compartment syndrome of the right leg with no evidence of necrotizing fasciitis. It is possible that the rapid course in this patient due to septicemia and multiorgan failure did not give time for the development of necrotizing fasciitis, but it clearly was not the initiating cause of this patient’s compartment syndrome. This is an example of the variability of presentation of* V. vulnificus* infections, a variability that appears to be due to the characteristics of both the bacteria and the host.

The lethal effect of *V. vulnificus* is mediated primarily by its secreted toxins, particularly the MARTX toxin. Therefore, along with third-generation cephalosporins, the use of antibiotics that block protein synthesis, such as doxycycline or minocycline (which bind to the bacterial 30s ribosomal subunit), are important in controlling the systemic effects of *V. vulnificus* [18]. This patient was diagnosed with a soft tissue skin infection and was initially started on vancomycin, cefepime, and clindamycin. Doxycycline was added about four to five hours later once the history of brackish water exposure was obtained. This strain of *V. vulnificus* was sensitive to both cefepime and doxycycline, but the protein-inhibiting effects of doxycycline are an important reason it is recommended as first-line therapy for known or presumed *V. vulnificus* infections.

The availability of rapid and inexpensive sequencing makes it easier to more fully characterize clinical and environmental* V. vulnificus *isolates. We used three methods to characterize the lethal strain of *V. vulnificus* in this patient. First, PCR was used to amplify a segment of the genome that was previously shown to discriminate between environmental (i.e., strains mainly from oysters or seawater) and clinical (i.e., strains from patients) isolates. This patient’s strain fit within the expected clinical PCR identification, but this approach provides no plausible mechanism to explain the differences in virulence. Next, the MARTX toxin was cloned and sequenced followed by sequence alignment with other known *V. vulnificus* strains. While this allowed us to demonstrate that this strain clustered geographically with other *V. vulnificus* strains from the Gulf Coast, it did not explain why this strain was so virulent in this patient. Lastly, whole genome sequencing was performed on the strain. Whole genome sequencing provides sequence information not only on the MARTX toxin but also on the entire bacterial genome and can identify coding and non-coding regions that might explain the variable clinical course of *V. vulnificus* infections. As shown in Figure 3, this strain was less related to the other *V. vulnificus* isolates than they were to each other. Interestingly, the group of vcgE isolates, which included a mix of isolates from clinical and environmental (oyster) sources, were highly related (18-38 SNPs) to each other, and were slightly more related to this patient’s strain (AFM1 (218-226 SNPs)) than the vcgC clinical isolates (234-256 SNPs). These results demonstrate the value of whole genome sequencing to define potential relationships among *V. vulnificus *isolates.

## Conclusions

This case underscores the severity of *V. vulnificus *sepsis and the urgency in considering *V. vulnificus* as a potential cause of gastroenteritis or soft tissue skin infections so that early therapy with appropriate antibiotics can be started. No evidence to date indicates that differences in genetic composition between environmental and clinical strains of *V. vulnificus* can explain the differences in clinical outcomes once these strains infect humans. Most sequencing studies have focused primarily on the open reading frame for the MARTX toxin; however, more intensive interrogation of *V. vulnificus* isolates using whole genome sequencing can determine whether sequences outside the MARTX toxin reading frame can explain the differences in virulence between strains in a way not currently understood. Better and more cost-effective whole genome sequencing, one beneficial result of the COVID-19 pandemic, may allow better characterization of *V. vulnificus* isolates, especially in cases where isolate sequences can be directly paired with clinical outcomes. This will require collaboration between the clinicians caring for these patients, the microbiology lab technicians who isolate the strains, and the basic scientists who can characterize the bacteria and its toxins at the molecular level. If there is no clear molecular pattern that identifies strains with increased virulence, then renewed attention on the clinical environment in which the strains are living or on the characteristics of susceptible hosts (particularly individuals with cirrhosis) would be warranted.
